# Longitudinal Photoreceptor Phenotype Observation and Therapeutic Evaluation of a Carbonic Anhydrase Inhibitor in a X-Linked Retinoschisis Mouse Model

**DOI:** 10.3389/fmed.2022.886947

**Published:** 2022-06-28

**Authors:** Meng Liu, Jingyang Liu, Weiping Wang, Guangming Liu, Xiuxiu Jin, Bo Lei

**Affiliations:** ^1^Zhengzhou University People's Hospital, Henan Provincial People's Hospital, Zhengzhou, China; ^2^Academy of Medical Sciences, Zhengzhou University, Zhengzhou, China; ^3^Henan Eye Institute, Henan Eye Hospital, Henan Provincial People's Hospital, Zhengzhou, China

**Keywords:** retinal degeneration, X-linked juvenile retinoschisis, carbonic anhydrase inhibitor, mouse, *Rs1*, ERG

## Abstract

**Purpose:**

To study the long-term photoreceptor changes and to evaluate the effects of topical application of a carbonic anhydrase inhibitor (CAI) in a mouse model of X-linked retinoschisis (XLRS).

**Methods:**

Conventional electroretinograms (ERGs) and dark-adapted 10-Hz flicker ERGs were recorded in control and *Rs1*^−/*Y*^ mice generated with CRISPR/Cas9. ON-pathway blocker 2-amino-4-phosphobutyric acid (APB) was injected intravitreally. Morphology was evaluated with histology and optical coherence tomography (OCT). Mice were treated with a CAI inhibitor brinzolamide eye drops (10 mg/ml) three times a day for 3 months. OCT and ERG findings at 1, 4, and 10 months were analyzed.

**Results:**

Negative ERGs and retinal cavities were evident in *Rs1*^−/*Y*^ mice. Both a-wave and b-wave amplitudes decreased with age when compared with age-matched controls. The APB-isolated a-wave (a′) amplitudes of *Rs1*^−/*Y*^ mice were reduced in all age groups. In dark-adapted 10-Hz flicker ERG, the amplitude-intensity curve of *Rs1*^−/*Y*^ mice shifted down. The thickness of ONL and IS/OS decreased in *Rs1*^−/*Y*^ mice. CAI reduced the splitting retinal cavities but didn't affect the ERG.

**Conclusions:**

In addition to post receptoral impairments, photoreceptor cells underwent progressive dysfunction since early age in *Rs1*^−/*Y*^ mice. Long-term CAI treatment improved the shrinkage of the splitting retinal cavity, while no functional improvement was observed.

## Introduction

X-Linked retinoschisis (XLRS) is the leading cause of vision loss in young men, accounting for approximately 5% of all childhood-onset inherited progressive retinal dystrophies (1). XLRS is caused by mutations in RS1 gene and affects males more than females due to its X-linked recessive inheritance (2). A hallmark of the XLRS is the splitting of the retina which frequently presents by ophthalmoscopy as a “spoke-wheel” pattern on the macula (3, 4). These cavities are readily observed via optical coherence tomography (OCT) (5).

Another hallmark of XLRS is negative electroretinogram (ERG) response. In most cases, the b-wave amplitude is disproportionately lower than the a-wave (6), and the b/a ratio reduces to 1.0 or less. The affected males exhibit normal or nearly normal a-wave (originates from photoreceptors) amplitudes suggesting relatively preserved photoreceptor functions, but substantially reduced b-waves (originates from bipolar cells), implicating defects at or beyond synaptic transmission.

The clinical phenotype of XLRS and the severity of clinical presentation vary at different ages. Young males with XLRS usually have diminished visual acuity at school age (7). Vision usually deteriorates during the first and second decades of life and stays relatively stable until the fifth or sixth decade (8). However, progressive morphology changes are noticeable during this period. The gradual shrinkage of the split cavities in the inner retina is often seen in most cases. More importantly, some patients presenting macular atrophy and loss of macular photoreceptors (9). Nevertheless, although impaired signal transmission beyond the photoreceptors has been extensively studied, mutation associated photoreceptor degeneration in this condition has barely been documented (10, 11). Thus, the first aim of this study was focused on the longitudinal photoreceptor morphology and function changes in a mouse model of XLRS. The importance of such a study is exaggerated by the fact that the RS protein is heavily expressed in the inner segment of photoreceptor and its role in this area has been overlooked.

Recent clinical observations studies have shown that carbonic anhydrase inhibitors (CAIs) may exert multiple benefits for XLRS including reduction of the cystic cavities and modest improvement of vision (12–15). In a relevant study, CAI acted on the membrane-bound carbonic anhydrase IV (CAI-IV) receptors on the retinal pigment epithelium (RPE), and acidified the extracellular space (16). It decreased subretinal space volume by increasing the fluid transport across the RPE through changing the extracellular pH gradients. In XLRS, because the decreased visual acuity was associated with the presence of cystic cavities, it was reasonable to predict that CAI may improve the visual function by reducing the size cysts (17). However, a short-term study on *Rs1*^−/*Y*^ mice denied therapeutic effect of CAI (18). To further confirm the potential beneficial roles of CAI in XLRS, we extended the study by a longitudinal observation in mice.

## Materials and Methods

### Generation of *Rs1^−/*Y*^* Mouse Model

The *Rs1* knockout mouse was custom designed in our lab with CRISPR/Cas9 technique. We selected *C57BL/6J* mice as the background strain and targeted *Rs1-201* (NM_011302.3) to design a specific sgRNA to lead Cas9 endonuclease to the target region, and made specific DSBs (double-stranded Breaks) into the genome of mouse followed by non-homologous end joining (NHEJ) repair pathway and excises all coding regions, gRNAs and Cas9 mRNA were transcribed *in vitro* and injected into the pronuclei of fertilized eggs of *C57BL/6J* mouse and implanted into surrogate mothers to obtain founders. Founders were identified by PCR genotyping and confirmed by DNA sequencing analysis. They were then bred with wildtype C57BL/6J to produce generation 1 (F1), and correct transmission of the mutation was identified by PCR genotyping and confirmed by DNA sequencing analysis. Hemizygous male (*Rs1*^−/*Y*^) and heterozygous female (*Rs1*^−/+^) F1 was bred with wildtype *C57BL/6J* mice to establish mouse colonies. The *Rs1*^−/*Y*^ mouse model was generated with assistance from Beijing Vital River Laboratory Animal Technologies Co. Ltd (Beijing, China). Animals were housed under 12 h light-dark cycle and given a standard chow diet. Animal care and use followed the guidelines formulated by the Association for Research in Vision and Ophthalmology (ARVO). Experimental designs and procedures were approved by the Ethics Committee of Henan Eye Hospital. Every effort was made to minimize animal discomfort and stress.

### PCR Genotyping

*Rs1*-knockout mice were screened by PCR amplification using tail DNA as the template, with two sets of oligonucleotide primers. One set (Forward: 5′TTAGCACATTCAGAAGAGGAGCGTA3′; Reverse: 5′-CAGTTTAAGGGAAACCTCACTATCCAC-3′) was designed to amplify the wild-type *Rs1* gene, with a product size of 350 bp. The other set primer (Forward: 5′ AGTACCATGCCATTTCAATCTCAACAA3′; Reverse: 5′ CAGTTTAAGGGAAACCTCACTATCCAC3′) was used to detect the mutant *Rs1* gene with a product size of 670 bp.

### Western Blot

Total retinal protein (10–50 μg) was loaded onto 10% SDS-PAGE gel. The protein was blotted onto a polyvinylidene difluoride (PVDF) membrane (Millipore, Burlington, MA, USA). Primary Rs1 antibody was used (1:1,000; Sigma-Aldrich, St. Louis, MO, USA). The bands were visualized with HRP-conjugated secondary antibody (Cell Signaling Technology, Beverly, MA, USA) and ECL detection reagents (Millipore).

### Intravitreal Injection of APB

2-Amino-4-phosphobutyric acid (APB) was purchased from Sigma-Aldrich (St. Louis, MO). Injection was performed according to a protocol described previously (19, 20). Briefly, the mice were anesthetized with an intraperitoneal injection with a 4% chloralhydrate solution. The pupils were dilated with 0.5% tropicamide drops for at least 10 min prior to injection. An aperture was made through the sclera, below the *ora serrata* with a 30-gauge needle, then a blunt 33-gauge Hamilton syringe was inserted through the aperture, avoiding damage the lens and making sure that a single 1 μl APB/PBS was injected into the vitreous under a dissecting microscope (Leica, DMR, Deerfeld, IL, USA). One μl APB (8.2 mM) was injected intravitreally into the right eyes of the mice, the contralateral eyes were injected with same volume of PBS as control. One drop of Levofloxacin Eye Drops (Cravit^®^ 0.5%) was applied topically after intravitreal injection. After injection, animals were given oxygen and were monitored in a warm recovery enclosure.

### Preparation and Application of CAI

Beginning at 1 month of age, brinzolamide eye drops (Azopt^®^ 10 mg/ml) were applied topically to the left eyes of *Rs1*^−/*Y*^ mice three times a day with an 8-h interval between applications. The contralateral eyes received instillation of PBS as control. Treatment was continued for 3 months until the mice reached 4 months of age. This treatment timeline permitted us to obtain a pre-treatment baseline (1-month of age), the effects during treatment (4-months of age), and the effect after discounting treatment (10 month of age). Care was taken to avoid spilling of the solution while maintaining the fixation of the animal for at least 1 min. Efforts have been made to ensure exclusive local absorption of the drug by the ocular tissue and to prevent systemic distribution because of self-grooming of the mice after treatment (18).

### ERG Assessment

ERG was recorded followed our previous protocols (21). After overnight dark adaptation, mice were anesthetized with intraperitoneal injection with a 4% chloralhydrate solution. Oxybuprocaine hydrochloride eye drops (Benoxil^®^ 0.4%) were applied for ocular surface anesthesia. Mice were placed on a warming pad to maintain body temperature near 38°C. The pupils were dilated with tropicamide eye drops 30 mins prior to recordings. Active electrodes were gently positioned on the center of the cornea. Needle electrodes were subcutaneously inserted into the back and the tail as reference and ground leads respectively. All procedures were performed under dim red light. Full-field ERGs were recorded with a visual electrophysiology system (RetiMINER, AiErXi Medical Equipment Co., Ltd., Chongqing, China). A series of stimulus intensities ranged from −3 to 1 log cd-s/m^2^ were applied for dark-adapted ERGs.

For the dark-adapted 10-Hz flicker ERG, the interval between the two consecutive flash trains was set at 200 ms (22). To increase the signal noise ratio, 10 signals were averaged.

### Histologic Evaluation

The mice were sacrificed by inhalation of excessive CO_2_. Eyes were enucleated and immersed in 4% paraformaldehyde/PBS (PFA/PBS) overnight (21). Fixed eyeballs were embedded in low melting temperature agarose (Sigma-Aldrich, St. Louis, MO) and sectioned. The ONL and INL thickness of inferior and superior retina were measured separately between 500 and 1,000 μm from the optic nerve head. H&E stained sagittal semithin sections including the optic nerve head were evaluated.

### OCT

#### OCT Image Collection

Fundus photography and OCT examinations were performed using a Micron IV retinal imaging system (Phoenix Research Labs, California, USA). The pupils were dilated with 0.5% tropicamide drops for at least 10 mins. The mice were anesthetized with intraperitoneal injection of 4% chloralhydrate solution. Lubricant Eye Gel (Gen Teal^®^ Tears) were applied on the corneal surface to keep the eyes moist. A view of the mouse retina was visible in the bright-field image. Forty OCT images were averaged to enhance the quality of the resulting image. The peripheral retina could be observed by changing the angle between the camera and the eye.

#### OCT Layer Thickness Measurements

The measurement area was chosen one optic head diameter away from the optic nerve. The thicknesses of the retinal layers were measured from captured images using Insight software (Phoenix Laboratories). As the OCT measurements were taken at the same distance to the optic nerve head, the data were not normalized to the total retinal thickness.

### Statistics

All data were presented as mean ± standard error of the mean (SEM). Statistical analysis was undertaken using the GraphPad Prism (GraphPad Prism Software, Inc., San Diego, CA, USA). ONL, INL, and (OS+IS) thickness at three different age groups were analyzed by one-way ANOVA followed by Bonferroni correction for multiple comparisons. The rest results were analyzed by unpaired *t test*. *p* < 0.05 was considered to be statistically significant.

## Results

### Animal Model

**Figure 1:**
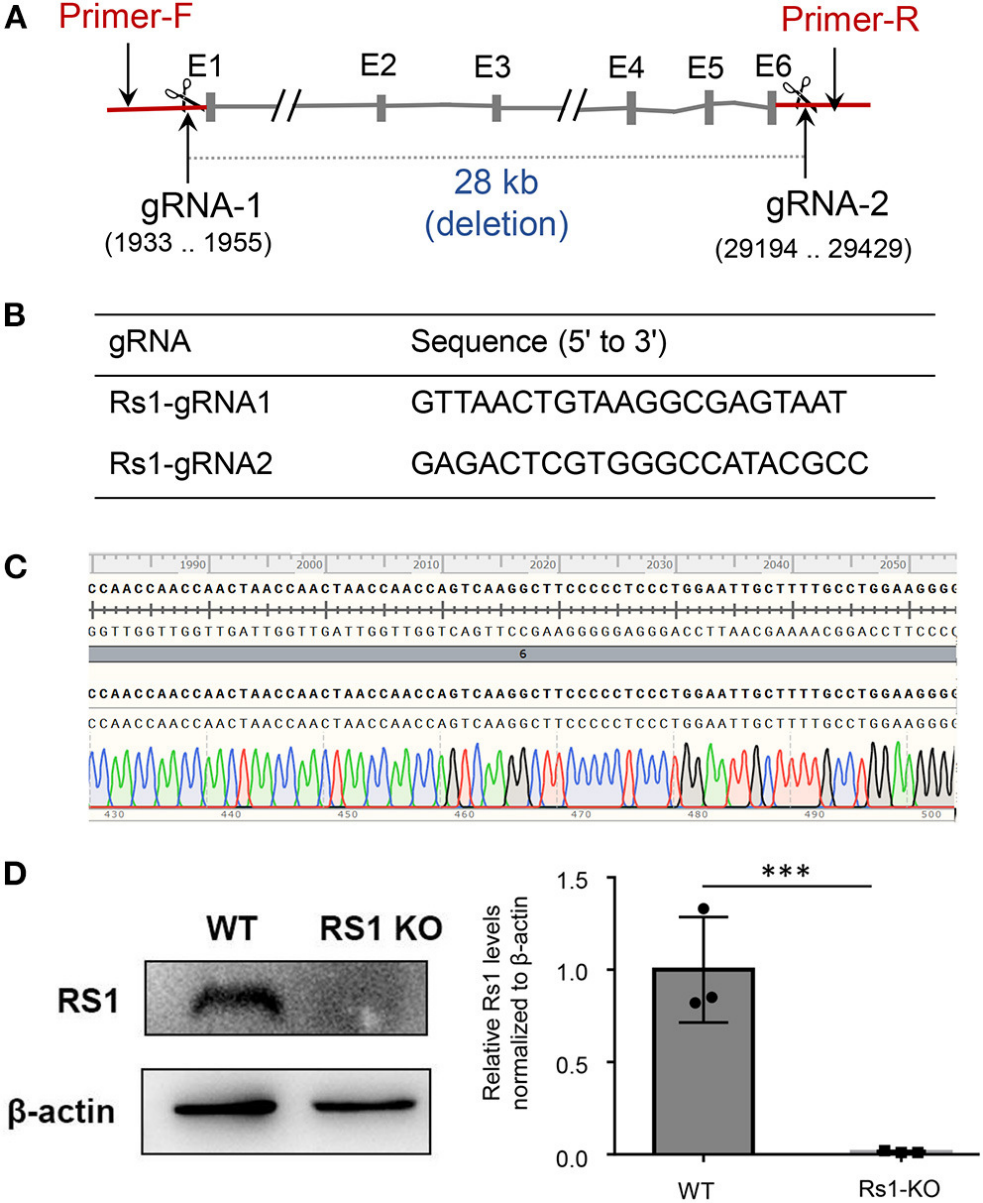
*Rs1*^−/*Y*^ mouse model generation and genotyping strategy. CRISPR/Cas9 genome editing system with two guide RNAs was used to generate the *Rs1*^−/*Y*^ mouse model. **(A)** In the targeting construct, all coding sequences of *Rs1* gene were deleted. Forward primer of genotyping located at the upsteam of gRNA-1 and reverse primer located at the downstream of gRNA-2. **(B)** gRNA sequencing of *Rs1* knockout mice. **(C)** DNA sequencing analysis revealed 28 kb deletion in one founder. **(D)** The absence of normal Rs1 protein in retinal extracts of *Rs1*^−/*Y*^ mice.

**Figure 2:**
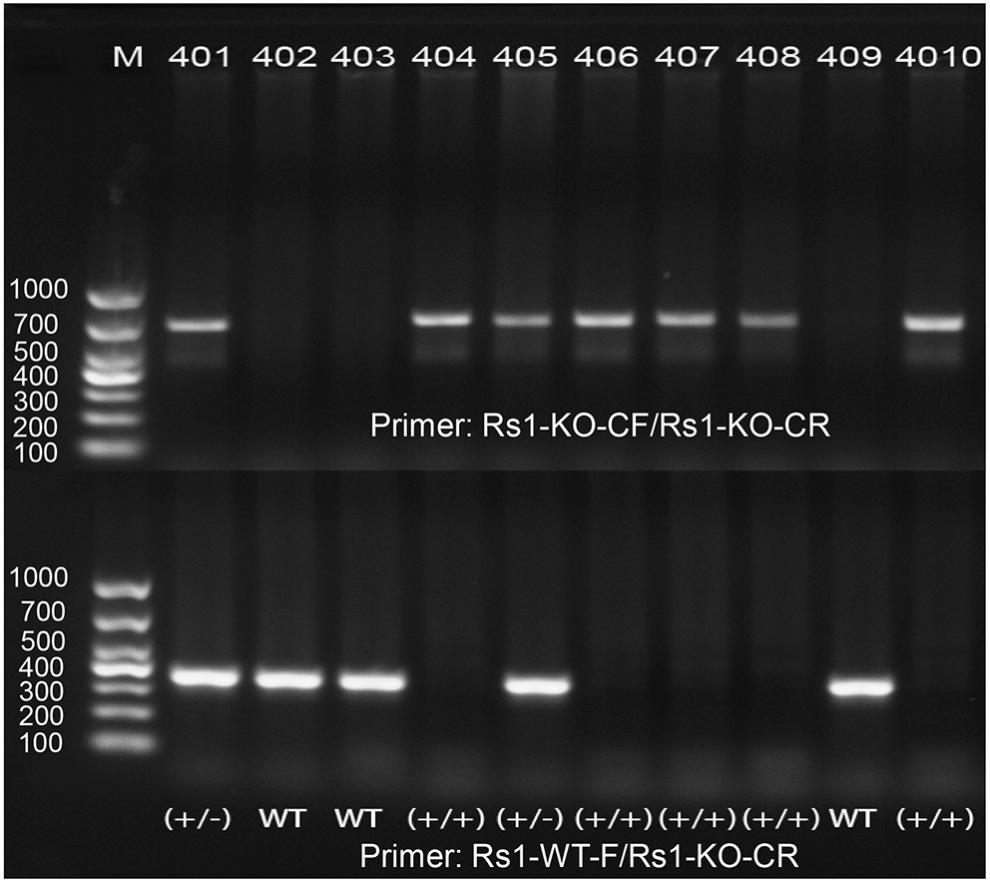
PCR amplification demonstrates the correct targeting of *Rs1*gene. One band of 350 bp was shown in WT, one band of 670 bp was shown in *Rs1*^−/*Y*^ mice, and both bands of 350 and 670 bp were shown in heterozygote.

Mouse *Rs1* Gene (Gene ID: 20147) is located on chromosome X and contains of three transcripts, of which *Rs1-201* is the most commonly used. We targeted *Rs1-201* to design a specific sgRNA to lead Cas9 endonuclease to the target region, and made specific DSBs (double-stranded Breaks) into the genome of mouse followed by non-homologous end joining (NHEJ) repair pathway and excises all coding regions. DNA sequencing confirmed deletion of all coding regions. PCR amplification of tail DNA and horizontal gel electrophoresis were performed to verify the successful construction of *Rs1*^−/*Y*^ mice. Absence of normal *Rs1h* protein was confirmed by Western blot and immunohistochemistry (IHC) (Figures 1, 2).

### Conventional Dark-Adapted ERG

**Figure 3:**
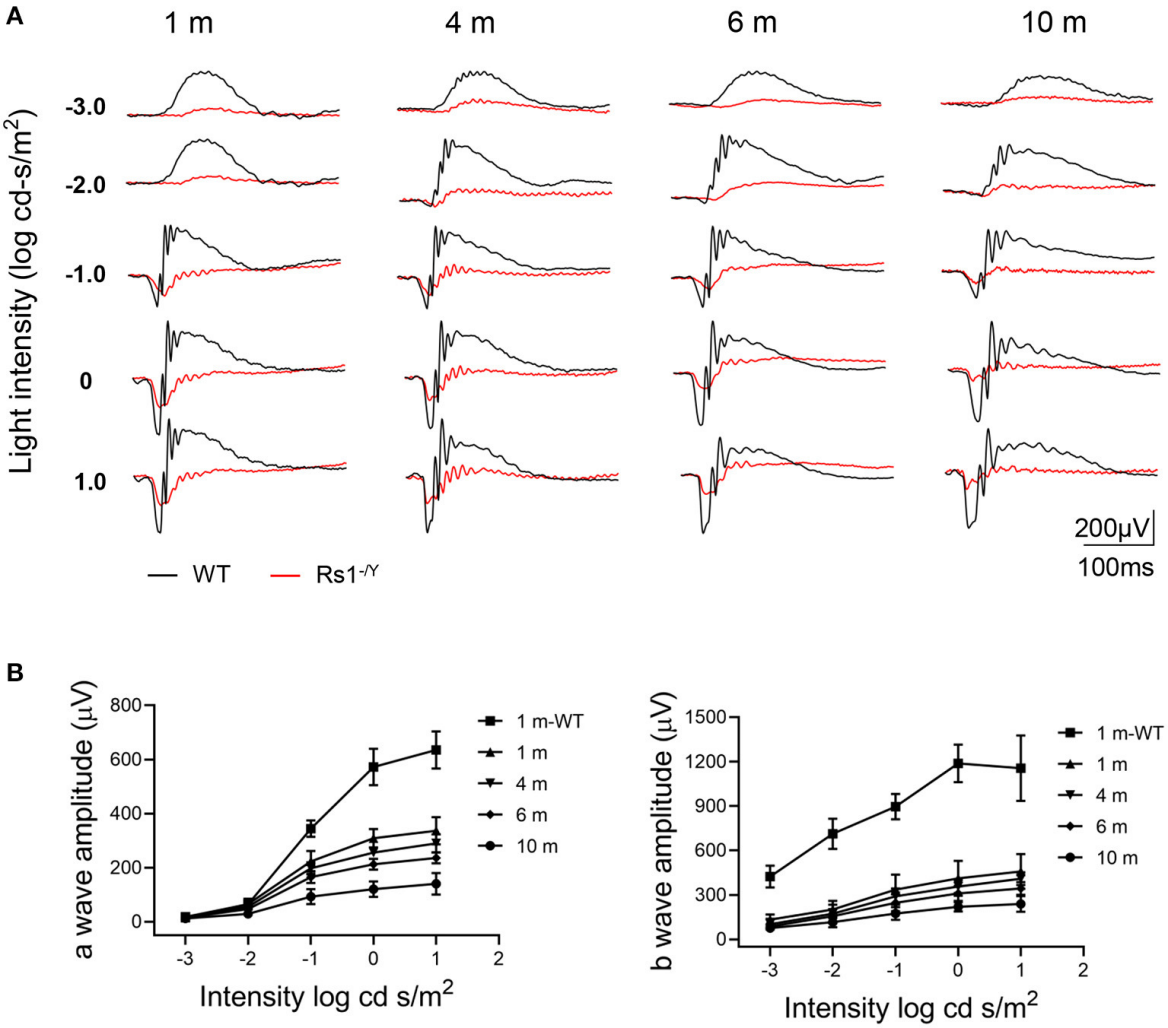
Representative ERG amplitude changes with age in *Rs1*^−/*Y*^ mice and age-matched WT mice. **(A)** A series of dark-adapted ERGs of *Rs1*^−/*Y*^ and WT mice of different ages (1, 4, 6, and 10 months). **(B)** a-Wave V-log I, b-wave V-log I. *n* = 9 at each age group. WT, wild type; ERG, electroretinogram.

Figure 3A showed a series of dark-adapted ERGs at 4 different ages (1, 4, 6, and 10 months). In *Rs1*^−/*Y*^ mice, both the a-wave and b-wave amplitudes (Figure 3B) throughout the stimulus intensity range decreased with age. At 1 month, the a-wave and the b-wave amplitudes reduced by 46% and 65% respectively, compared with age-matched control WT mice at 0 log cd.s/m^2^ stimulus intensity (Table 1).

**Table 1:** a-Wave and the b-wave amplitudes (0 log cd.s/m^2^).

	**a-wave**	**b-wave**	***n***
	**Mean ±SE (μV)**	**Mean ±SE (μV)**	
*Rs1^−/*Y*^* (1 m)	309 ± 11	411 ± 39	9
WT (1 m)	572 ± 22	1,188 ± 42	9

**Figure 4:**
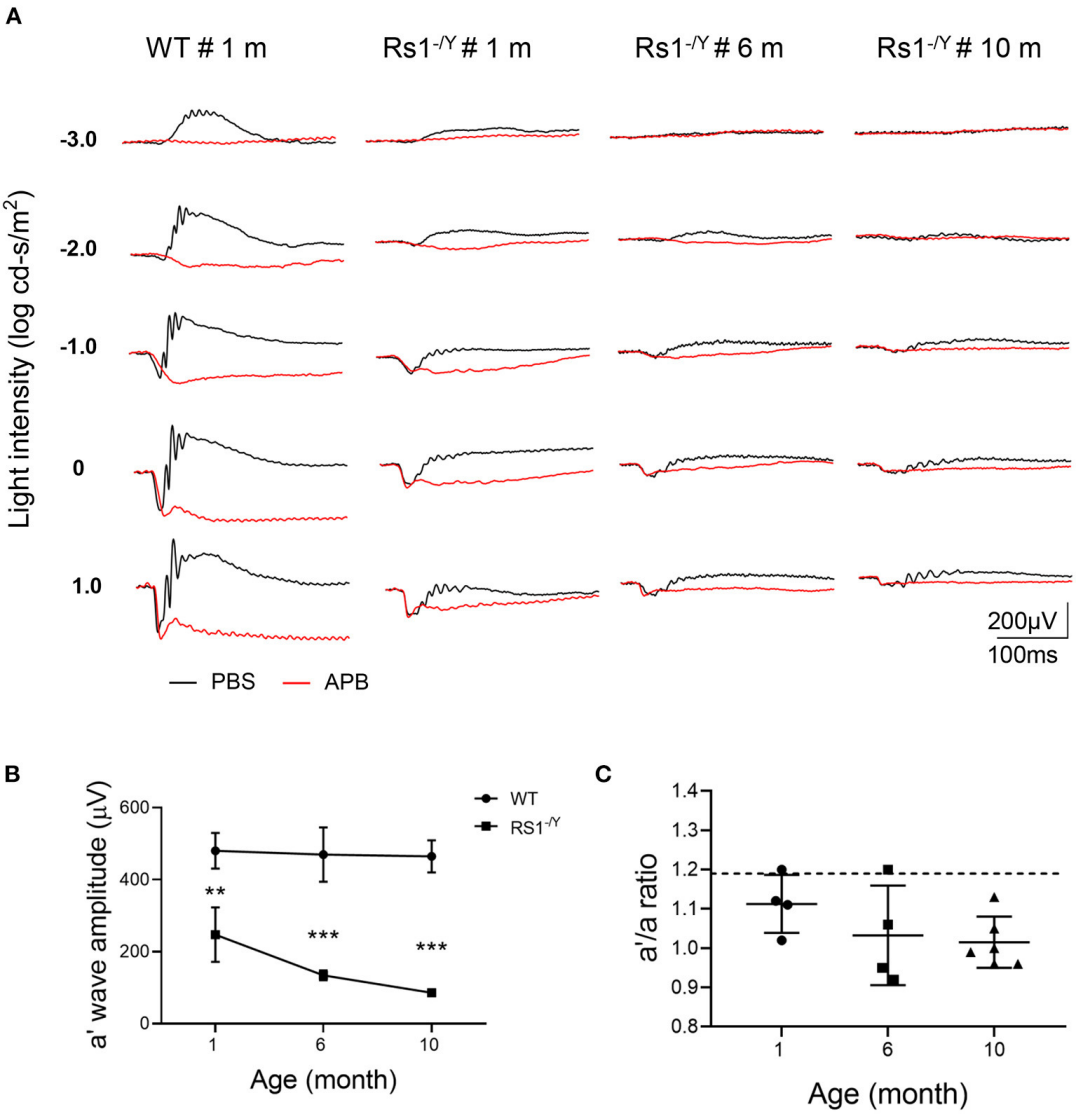
The effect of APB on the dark-adapted ERG of *Rs1*^−/*Y*^ mice and and age-matched WT mice. **(A)** The black traces show ERGs of the PBS-injected control eyes and the red traces show ERGs of the APB injected eyes. In the APB-injected eye, the a-wave remains but the b-waves are absent. **(B)** Left: The a′ wave amplitude changes with age in *Rs1*^−/*Y*^ mice of 1, 6, and 10 month old. ERG stimulus, 0 log cd-s/m^2^. **(C)** Right: a′/a ratio in *Rs1*^−/*Y*^ mice of three different ages (1, 6, and 10 months). Dashed lines: the a′/a ratio mean ± SE of WT: 1.190 ± 0.127 *n* = 5. a′ wave: the negative wave in APB injected eye; APB, 2-amino-4-phosphobutyric acid. *Rs1*^−/*Y*^ results compared to WT using Student's t-test: ^***^, *P* < 0.001.

Because of the interference of b wave, it's insufficient to evaluate photoreceptor function just by the a-wave amplitude. For better understanding of photoreceptor function of *Rs1*^−/*Y*^ mice, APB was injected intravitreally to abolish the b-wave. The b-wave of the dark-adapted ERG is dominated by ON bipolar cell responses which can be excluded by APB. However, APB does not eliminate OFF bipolar cell activity, which can be eliminated by PDA. Nevertheless, the OFF-pathway contribution was proved to be minimal in mouse a-wave (22). Therefore, the residual a′ wave reflected purely the response of photoreceptor cells. About 90 mins after the injection, the dark-adapted ERG b-wave was eliminated (Figure 4), and the a′ wave was dominant at high intensities. The a′ wave amplitude of *Rs1*^−/*Y*^ mice (0 log cd-s/m^2^) decreased significantly compared with age-matched WT mice (Table 2).

**Table 2:** a' wave of different age groups (0 log cd.s/m^2^).

	**1 m**	**6 m**	**10 m**
*Rs1^−/*Y*^*	247 ± 31 (*n* = 6)	134 ± 7 (*n* = 4)	86 ± 4 (*n* = 5)
WT	480 ± 29 (*n* = 3)	470 ± 44 (*n* = 3)	465 ± 26 (*n* = 3)

And the a′ wave also declined with age. Furthermore, the a′/a ratio, which reflected the relative potential of light elicited photoreceptor response, also declined in *Rs1*^−/*Y*^ mice when compared with WT mice. The ratio also decreased with age, further suggesting that the photoreceptor function of *Rs1*^−/*Y*^ mice declined as the disease progresses.

### The Dark-Adapted 10-Hz Flicker ERG

**Figure 5:**
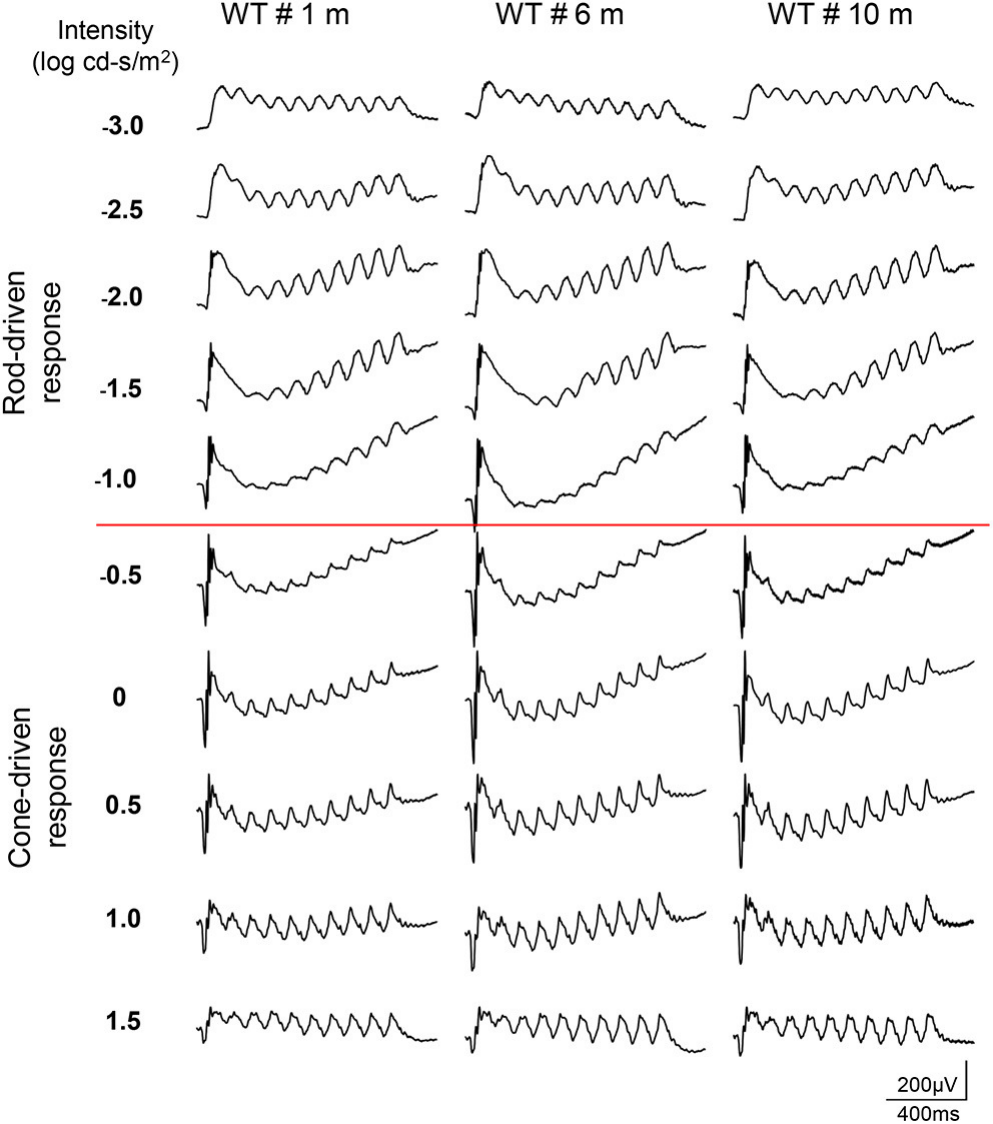
Dark-adapted 10-Hz flicker ERGs elicited with a series of light intensity in WT mice of different ages.

**Figure 6:**
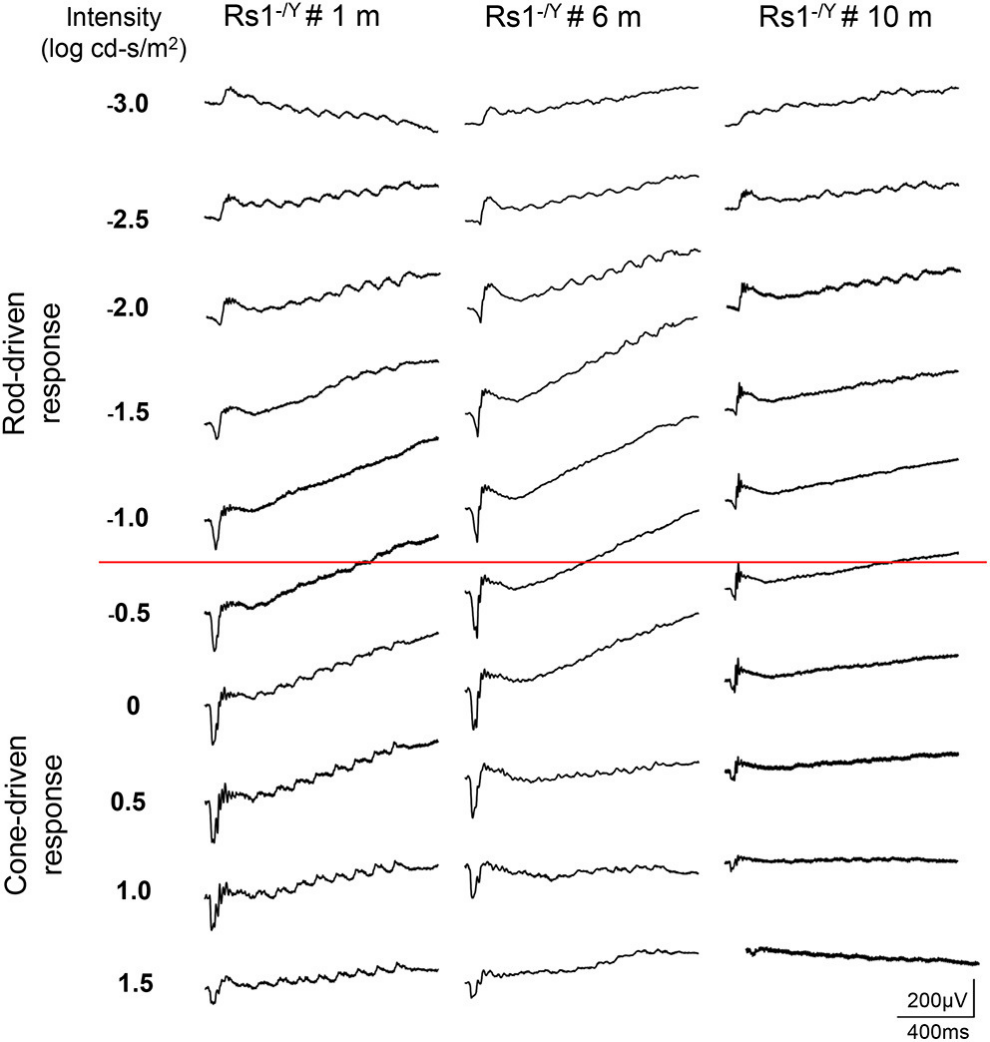
Dark-adapted 10-Hz flicker ERGs elicited with a series of light intensity in *Rs1*^−/*Y*^ mice of different ages.

The dark-adapted flicker ERG is practical for evaluation of rod-and cone-driven responses simultaneously. Dark-adapted 10-Hz flicker ERGs of WT, and *Rs1*^−/*Y*^ mice at 3 different ages were shown (Figures 5, 6), and Figure 5 showed the amplitude-intensity profiles (*n* = 3 in each age group). There were two peaks in the middle and high light intensities in the WT mice, with the first representing rod-driven and the second representing cone-driven responses.

**Figure 7:**
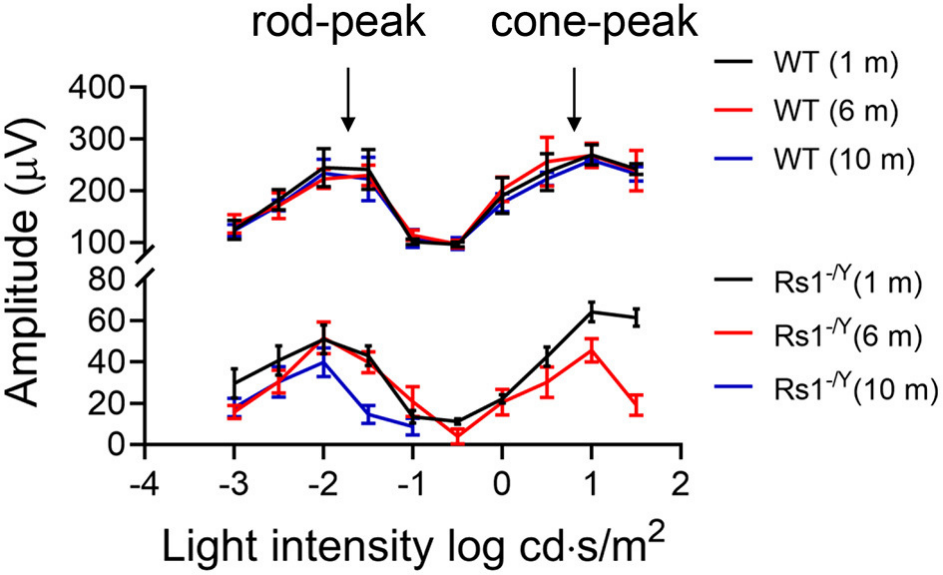
Dark-adapted 10-Hz flicker ERG response amplitude-intensity profiles (*n* = 3 at each age group).

In 1 month old *Rs1*^−/*Y*^ mice, both the rod- and the cone-driven responses existed, while the amplitude-intensity curve shifted down significantly. The curve of the 6-month-old *Rs1*^−/*Y*^ mice was similar to the curve at 1-month-old, except for a lower of the cone-driven response. In 10-months-old group, the rod-driven response still existed although significantly declined, but the cone-driven response was not detectable, suggesting the cone system was more vulnerable than the rod system in this model Figure 7.

### Morphological Abnormalities

**Figure 8:**
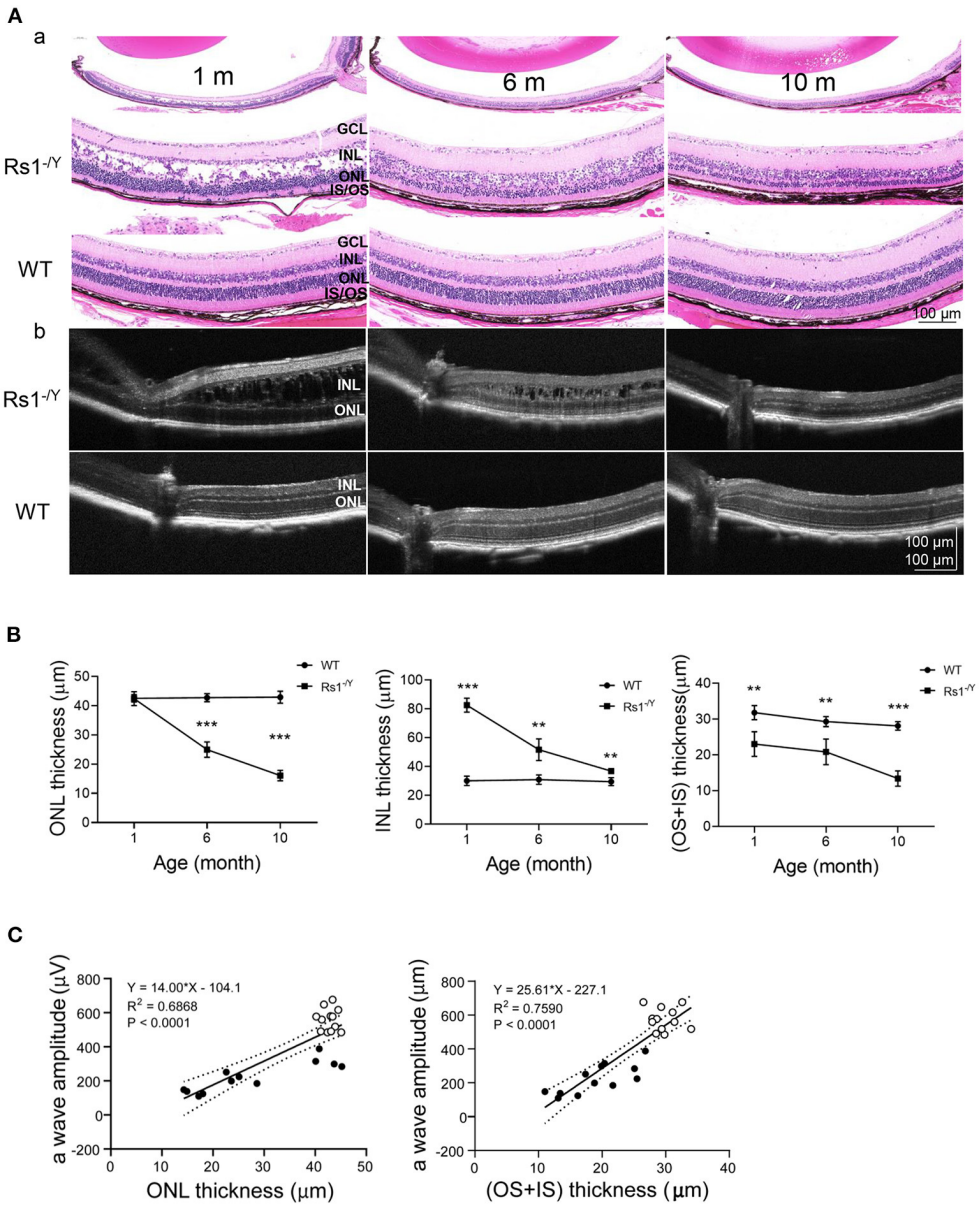
Morphology changes in *Rs1*^−/*Y*^ mouse and age-matched WT mice. **(A)** Representative sections from retinas of 1, 6, and 10-month-old *Rs1*^−/*Y*^ mice and age-matched WT mice and retinal images obtained from OCT were used to evaluate the retinal morphology at three different ages. **(B)** The thickness of the ONL, INL, OS+IS 300 μm away from the optic nerve was measured (*n* = 4 at each age group). **(C)** a-Wave amplitude vs. OS+IS thickness. *Lines*: linear regression (*R*^2^ = 0.7590) and 95% confidence intervals. a-Wave amplitude vs. ONL thickness. *Lines*: linear regression (*R*^2^ = 0.6868) and 95% confidence intervals, *P* < 0.0001; ONL, outer nuclear layer; INL, inner nuclear layer; OS, outer segment; IS, inner segment. *Rs1*^−/*Y*^ results compared to WT using Student's t-test: ^*^, *P* < 0.05; ^**^, *P* < 0.01; ^***^, *P* < 0.001.

We monitored the morphological changes of *Rs1*^−/*Y*^ mice at three different ages (*n* = 4 in each age group) by histology analysis and OCT (Figure 8). Large cavities within the INL were the most striking in 1-month-old Rs1^−/Y^ mouse retina, and the splitting cavities gradually collapsed and shrunk at 6 and 10 months. Thickness of the retinal layers was measured on histologic sections. At 1 month, the thickness of ONL was not significantly different from WT, while the structure of the ONL was disrupted with some nuclei were displaced into the OPL and the inner segment layers. The ONL thickness declined rapidly at 6 and 10 months. The INL thickness of 1-month-old *Rs1*^−/*Y*^ mice was larger than WT, due to the cavities within INL. With the subsequent collapse of splitting cavities, the INL became thinner gradually, but it was still thicker in *Rs1*^−/*Y*^ mice than the age-marched WT. The thickness of OS+IS in *Rs1*^−/*Y*^ mice decreased compared with WT at 1 month, and continued to diminish at 6 and 10 months (Figure 8B). OCT images of WT retina showed normal organized lamellar structure while the *Rs1*^−/*Y*^ mouse retina showed abnormalities corresponding to those observed in histology sections, and these changes were seen across the retina from center to periphery.

### CAI Treatment

**Figure 9:**
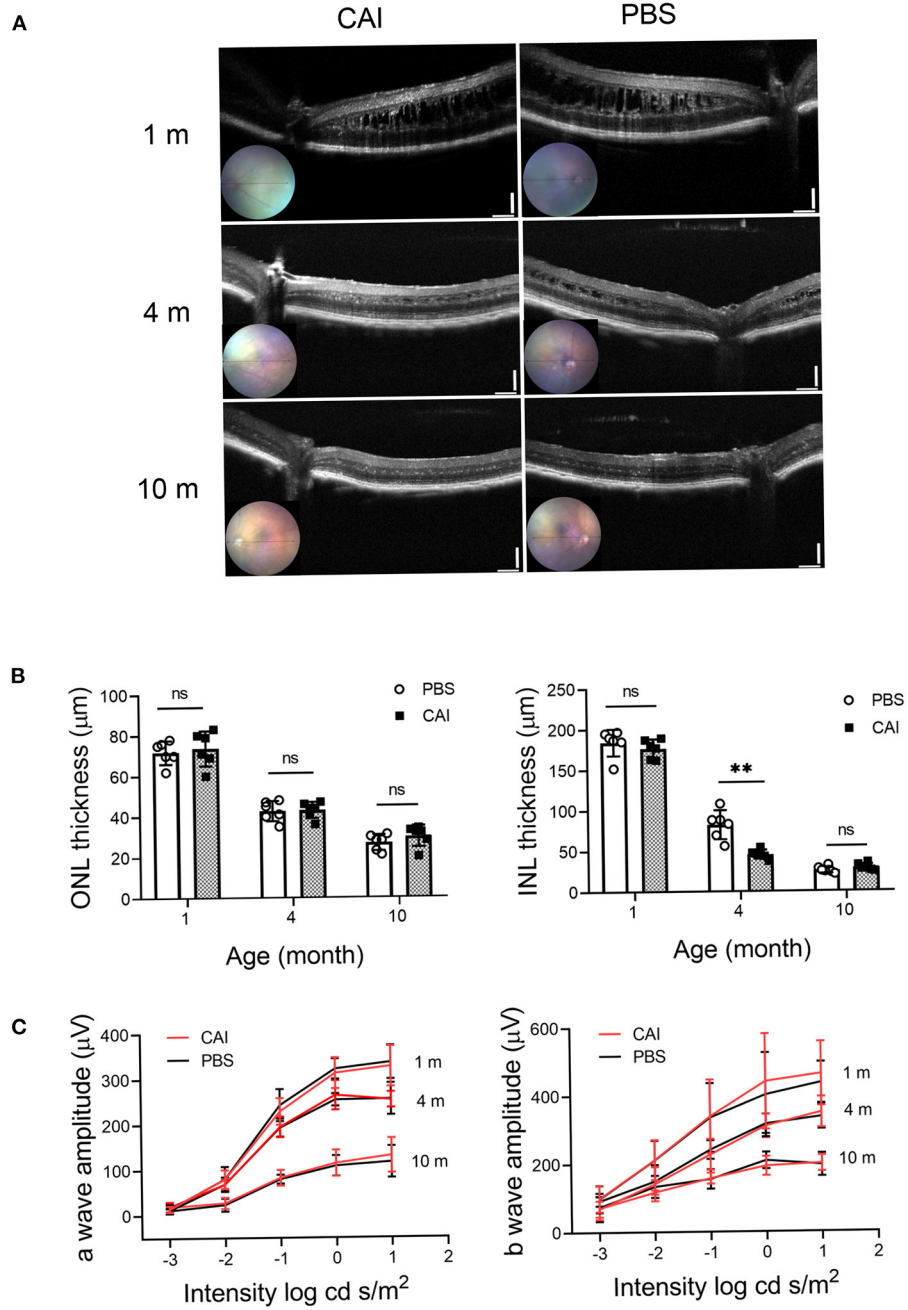
Structural and functional changes after CAI treatment in *Rs1*^−/*Y*^ mice retina were evaluated by OCT and ERG. **(A)** Retinal images obtained from OCT in living animals were used to evaluate the retinal morphology at 3 different ages. From 1 month, the left eyes of *Rs1*^−/*Y*^ mice were treated with brinzolamide eye drops (10 mg/ml) for 3 continuous months, the contralateral eyes were treated with PBS as control. OCT were performed at 1, 4, 10 months of age. **(B)** ONL and INL thickness were measured from 200 to 1,200 μm inferior and superior to the optic nerve (*n* = 6 at each time point). **(C)** Functional changes by CAI treatment in *Rs1*^−/*Y*^ mice retina were evaluated by ERG. The CAI treated eyes were compared with the control eyes using Student's *t*-test: ^***^, *P* < 0.001. CAI, carbonic anhydrase inhibitor; OCT, optical coherence tomography.

We tested the potential beneficial effects of long-term CAI treatment in the *Rs1*^−/*Y*^ mouse retina. The left eyes of 1-month-old *Rs1*^−/*Y*^ mice were treated with brinzolamide eye drops (3 times a day for 3 consecutive months), while the right eyes were treated with PBS as control. For a detailed evaluation of retinal function and structure, ERGs and OCT were performed at three time points (1, 4, 10 months of age) (*n* = 6 in each age group). At 1 month of age, the laminar structure of *Rs1*^−/*Y*^ mice was disrupted, and the thickness of ONL, INL showed no difference between the two eyes. After 3 months' treatment, the splitting cavities of the treated eyes were smaller, and the INL thickness decreased compared with the control eyes, while no difference in ONL thickness was observed between the two eyes. The cavities in INL continued to reduce and disappeared at the age of 10 months, but the thickness of ONL and the INL showed no difference between the two eyes. Additionally, the amplitudes of ERG a- and b-wave were similar between the treated and control eyes at all three time points (Figure 9).

## Discussion

Photoreceptors, including rods and cones, and bipolar cells are the first two order neuros in the retina. As all the three types of cells express RS1 protein, causative mutations in *RS1* gene may induce morphologic and functional damages to all these cells. However, because the most striking features of XLRS are the cystic cavities in the INL and the negative ERG waveform, both of which are convictive indicators of impairment in the inner retina, the photoreceptor impairment has relatively been overlooked. However, increasing studies reported a reduction in ERG a-wave of young XLRS patients (23, 24), indicating photoreceptor degeneration may be more prevalent in XLRS than previously thought.

Initial evidence of abnormal cone system function came primally from photopic ERG responses recorded in patients with XLRS: Alexander et al. reported that high-frequency response attenuation of the ERG in XLRS indicated abnormalities of the photoreceptors and depolarizing bipolar cells (DBCs) (25, 26). Multifocal electroretinogram (mfERG) revealed that the cone-mediated retinal responses were more impaired in the central than peripheral retina of XLRS patients (27). A recent study that re-examined photoreceptor and post-receptor ERG responses found smaller a-wave amplitudes in some young XLRS patients (23). In addition, some XLRS animal models also showed decreased a-wave amplitude at early age. For example, Liu's group demonstrated several disease phenotypes which presented at early ages in three *Rs1* mutant mouse models. Each model developed intraretinal schisis with OCT and showed early abnormalities in outer retina neurons with immunohistochemical analysis. In consistent with the morphological changes, the decrease of ERG b-wave amplitude was more prominent than the a-wave, but the a-wave was already abnormal at P15 and remained decreased till later stage (28). These findings indicated that although the major defects were in the inner retina, outer retina photoreceptor degeneration could present concurrently. In this study, we also found that a-wave reduced significantly compared with age-matched WT mice, and underwent a processive decline over time. At the same time, we found the cone system function of *Rs1*^−/*Y*^ mice was more vulnerable than the rod system. To rule out the interference of the second order neurons, intravitreal injection of APB was applied to eliminate the b-wave. The residual a′ wave were presumably purely generated from the photoreceptors. We found that the a′ wave amplitude decreased compared with age-matched WT mice at all three age groups, and displayed a decreasing trend with age. This provided further evidence that the *Rs1*^−/*Y*^ photoreceptor suffered from degeneration starting at early age and underwent progressive decline. However, this phenotype appeared more severe at early stage in *Rs1*^−/*Y*^ mice than in most XLRS patients. It was reported that decreased a-wave amplitude was observed in about 1/3 of XLRS patients (29). Because photoreceptors are not regenerable, it is of great importance to evaluate their function and structure to predict the prognosis of emerging interventions. Understanding the status of photoreceptors may raise significant concerns to these crucial cells and encourage early treatment for this condition.

Except for the split cavities, abnormalities in the outer retina were also observed by OCT examinations in patients with XLRS (30, 31). All the 7 XLRS mouse models together with the *Rs1*^−/*Y*^ rat model presented different rates of retinal degeneration (32–37). Our *Rs1*^−/*Y*^ mice displayed similar inner retinal morphology changes and progressive loss of photoreceptors which led to a markedly reduced ONL thickness. Good corrections between the a-wave amplitude and the ONL, OS+IS thickness may partially explain the progressive reduction in overall ERG response, as has been found for other animal models with primary photoreceptor degeneration (38). It has been suggested that RS1 modulates cellular homeostasis by binding to Na/K-ATPase (39, 40). RS1 could affected the Na/K-ATPase-regulated mitogen-activated protein kinase /extracellular-signal-regulated kinase (MAPK/ERK) signaling cascade and Ca^2+^ signaling. These studies provided evidence that RS1 deficiency might be one of the initial steps that triggered XLRS pathology in retinal degeneration, rather than just the acknowledged adhesive interactions on retinal structure in inner retina.

Both XLRS patients and animal models showed natural evolution of the cystic cavities, which typically collapsed overtime. Since cystic presence has been linked to decreased visual acuity (41), it was conjectured that CAI might improve the acuity and decrease the later-onset atrophy in XLRS, by reducing fluid accumulation in the cavities. We found CAI reduced cystic cavity volume in *Rs1*^−/*Y*^ mice, while no functional improvement was detected in term of ERG responses, which were comparable to the results of clinical trials in human XLRS subjects (42–44). The reasons that the discordance between the structure and function after CAI application remained unknown. One interpretation could be the progressive structural deterioration presenting in the outer retina, including IS/OS, gap junctions, and synapses, which are indispensable for the generation of ERG. However, those damages could not be reversed by CAI treatment alone. It could also be due to the early nerve damage might occur before the treatment began. Therefore, earlier CAI intervention starting from eye opening might be more effective. Besides, the effect of selective inhibitors on the enzymatic isoforms expressed in the retinal/eye tissues deserves further research. Nevertheless, accelerated shrinkage of the splitting cavity by CAI could be helpful in restoring retinal anatomy, prolong the therapeutic time window for gene therapy, and thereby improve the therapeutic effect (45, 46).

In summary, we documented that photoreceptor cells underwent progressive dysfunction since early stage in an XLRS mouse model. And the cones are more vulnerable to the genetic defect than the rods. In addition, long-term CAI treatment was beneficial in promoting the shrinkage of the splitting cavity in *Rs1*^−/*Y*^ mice, while functional improvement was not observed. Nevertheless, CAI might be an adjunct medication to the emerging gene therapy for XLRS.

## Data Availability Statement

The original contributions presented in the study are included in the article/supplementary material, further inquiries can be directed to the corresponding author/s.

## Ethics Statement

The animal study was reviewed and approved by Ethics Committee of Henan Eye Hospital, Henan Provincial People's Hospital.

## Author Contributions

BL contributed to the conceptualization, design, and outline of this review. ML conducted the experiments and prepared the draft of the manuscript. JL, WW, and GL helped in the experiment procedures. BL, XJ, and ML contributed to analyzing data, revision, and editing. All authors have read and approved the final manuscript.

## Funding

This work was supported by grants from the National Natural Science Foundation of China (81770949 and 82071008), Key Technologies Research and Development Program of Henan Science and Technology Bureau (212102310307), and Medical Science and Technology Program of Health Commission of Henan Province (SBGJ202003014 and LHGJ20200070).

## Conflict of Interest

The authors declare that the research was conducted in the absence of any commercial or financial relationships that could be construed as a potential conflict of interest. The reviewer XZ declared a past co-authorship with the author BL to the handling editor.

## Publisher's Note

All claims expressed in this article are solely those of the authors and do not necessarily represent those of their affiliated organizations, or those of the publisher, the editors and the reviewers. Any product that may be evaluated in this article, or claim that may be made by its manufacturer, is not guaranteed or endorsed by the publisher.
